# Can Additional Homeopathic Treatment Save Costs? A Retrospective Cost-Analysis Based on 44500 Insured Persons

**DOI:** 10.1371/journal.pone.0134657

**Published:** 2015-07-31

**Authors:** Julia K. Ostermann, Thomas Reinhold, Claudia M. Witt

**Affiliations:** 1 Institute for Social Medicine, Epidemiology and Health Economics, Charité - University Medical Center, 10117 Berlin, Germany; 2 Institute for Complementary and Integrative Medicine, University of Zurich and University Hospital Zurich, Zurich, Switzerland; 3 University of Maryland School of Medicine, Center for Integrative Medicine, Baltimore, Maryland, United States of America; University of Geneva, SWITZERLAND

## Abstract

**Objectives:**

The aim of this study was to compare the health care costs for patients using additional homeopathic treatment (homeopathy group) with the costs for those receiving usual care (control group).

**Methods:**

Cost data provided by a large German statutory health insurance company were retrospectively analysed from the societal perspective (primary outcome) and from the statutory health insurance perspective. Patients in both groups were matched using a propensity score matching procedure based on socio-demographic variables as well as costs, number of hospital stays and sick leave days in the previous 12 months. Total cumulative costs over 18 months were compared between the groups with an analysis of covariance (adjusted for baseline costs) across diagnoses and for six specific diagnoses (depression, migraine, allergic rhinitis, asthma, atopic dermatitis, and headache).

**Results:**

Data from 44,550 patients (67.3% females) were available for analysis. From the societal perspective, total costs after 18 months were higher in the homeopathy group (adj. mean: EUR 7,207.72 [95% CI 7,001.14–7,414.29]) than in the control group (EUR 5,857.56 [5,650.98–6,064.13]; p<0.0001) with the largest differences between groups for productivity loss (homeopathy EUR 3,698.00 [3,586.48–3,809.53] vs. control EUR 3,092.84 [2,981.31–3,204.37]) and outpatient care costs (homeopathy EUR 1,088.25 [1,073.90–1,102.59] vs. control EUR 867.87 [853.52–882.21]). Group differences decreased over time. For all diagnoses, costs were higher in the homeopathy group than in the control group, although this difference was not always statistically significant.

**Conclusion:**

Compared with usual care, additional homeopathic treatment was associated with significantly higher costs. These analyses did not confirm previously observed cost savings resulting from the use of homeopathy in the health care system.

## Introduction

Homeopathy is frequently used by patients in the German healthcare system. A cross-sectional survey representative of the population indicated that 60% of all 1503 persons interviewed had used homeopathy [1] and that 1.97% (n = 7030) of all German physicians specialize in homeopathy [2]. Homeopathy originates from a time when medicine would often do more harm than good [3]. Homeopathy involves taking an elaborate patient history and prescribing homeopathic remedies based on individual case histories. Homeopathy is based on the principles of curing like with like and of diluting and shaking a substance to enhance its effectiveness [4,5]. Substances may be diluted up to a concentration in which no molecule of the original substance can be found in the dilution [6]. Apart from Germany, homeopathy is popular among many people around the globe, especially in Europe [7]. Its popularity stems, among other causes, from the often extensive consultation time that homeopathic physicians provide, the absence of adverse effects and the belief in its effects [8,9]. Despite the current lack of valid explanations for the mechanism of action of highly diluted homeopathic remedies [6,10], homeopathy is reimbursed by more than 80 statutory health insurance companies in Germany [11]. In addition to the competition for new insurants, the potential for cost savings is often used by statutory health insurance companies as a relevant argument for reimbursing homeopathy. This cost-saving potential has been supported by several studies that compared homeopathy with conventional medicine [12–14]. However, our own health economic evaluations did not show a consistent picture. We observed no differences in costs [15] or additional costs [16,17] in the homeopathic group compared to conventional care depending on the setting or diagnosis.

A recent systematic review that summarized the available literature on cost-effectiveness provided no distinct conclusion [7]. Homeopathy is reimbursed by German statutory health insurance companies within the context of integrated care contracts [11], and patients can be enrolled in integrated care contracts by their physicians. Integrated care contracts are both voluntarily and free of charge for the patients, participating physicians receive an additional lump sum compensation. The aim of the integrated care contract homeopathy is to enable cooperation between physicians and pharmacies to coordinate patients’ homeopathic treatments.

The objective of this study was to compare the health care costs of insured persons who used additional homeopathic treatment within an integrated care contract framework with the costs for patients who used only usual care through one of the largest statutory health insurance companies in Germany.

## Methods

### Study design and participants

Health care cost data were compared for patients who used additional homeopathic treatment within the integrated care model homeopathy (homeopathy group) and those who used only usual care (control group) based on claims data from one of the largest German statutory health insurance company (Techniker Krankenkasse, TK (www.tk.de)). For this analysis, patients belonged to the homeopathy group if they subscribed to the integrated care contract in 2011 and if they were continuously insured through the TK for the observational period (12 months before and 18 months after subscription to the integrated care contract), regardless of whether they used homeopathy during the study period. The start date of the observation period for the homeopathy group was the start date of the integrated care contract subscription, which indicated the commencement of homeopathic treatment. The control group was drawn from a sample of all TK-insured persons who did not subscribe to the integrated care contract, who were continuously insured through the TK for the observational period, who contacted a physician during the first three months of the observation period, and who were successfully matched with a homeopathy user based on propensity scores. The start date of the observation period for the controls was determined as the start date of the integrated care contract subscription of the homeopathy users (notional start date). In addition to comparing health care costs across diagnoses between groups, data were also analysed for specific confirmed diagnoses: depression (ICD-10 F32), migraine (G43), allergic rhinitis (J30), allergic asthma (J45), atopic dermatitis (L20), and tension headache (R51). We followed the guidelines for secondary data analyses. Patient claims data were pseudonymised by the statutory health insurance. We had no key for de-identifying the pseudonymous data. The study was approved by the Ethics Committee of the Charité—Universitätsmedizin Berlin (EA2/121/12).

### Propensity score matching

Because any person insured through the TK could potentially subscribe to the integrated care contract, a randomized controlled study design was not possible. To minimize selection bias and to balance baseline characteristics between groups, homeopathy users and controls were matched in a 1:1 ratio using propensity scores [18]. Propensity scores for the outcome ‘user of the integrated care contract’ were calculated for participants in both groups using the following covariates: sex (male/female), age (continuous), comorbidities (disease present, yes/no), cumulative different unit costs one year prior to the study period (continuous), length of stay in a hospital (continuous), days of sick leave (continuous) and statutory sick pay costs (continuous), duration of outpatient rehabilitation (continuous), level of care intensity (‘1’, ‘2’, ‘3’ or ‘3 plus’), disease-management-program participation (yes/no), usage of GP-centred care (yes/no) and population density (inhabitants per square kilometre, continuous). Controls were matched to homeopathy users using the respective propensity scores with a caliper width equal to 0.25 of the standard deviation of the logit of the propensity score, which resulted in a good matching quality. Matching was performed four times for each of the four three-month periods (quarters) in 2011 to prevent seasonal effects between the groups. Matching was first performed for each of the abovementioned diagnoses. That is, a control could be matched to a homeopathy user with a confirmed diagnosis if the control had the same confirmed diagnosis in the same three-month interval during which the homeopathy user subscribed to the integrated care contract. Because the index date for the controls was unknown before the matching was performed, cumulated costs for controls for the propensity score were calculated using the middle of the quarter for which the matching was performed as the index date. For each of the four matching groups, claims data were analysed for a total of 30 months, 12 months before and 18 months after the start date of the integrated care contract. Data were therefore analysed from January 2010 to June 2013.

### Economic analysis

For the primary analysis, costs were analysed from the societal perspective across all diagnoses. For the secondary analysis, costs were analysed for the six pre-specified diagnoses. Additionally, data were analysed from the statutory health insurance perspective. Productivity loss calculations for the societal perspective were based on the human capital approach using a daily gross mean income of EUR 239.20 with a cut-off period of six weeks [19]. Costs were not discounted because of the short observation period. Costs were calculated 12 months prior to the start of integrated care contract (or the notional start date for controls respectively) and 18 months after the start of the integrated care contract. To evaluate possible sources of cost utilization in outpatient care, outpatient costs were divided into costs from the homeopathic physicians who participated in the integrated care contract and costs from other physicians. Controls could have been treated by a homeopathic physician, but only outside of the integrated care contract. To address interpretation issues, negative cost values in the data were set to zero.

### Statistical analysis

For the primary end point, an analysis of covariance (ANCOVA) with cumulative costs of month -12 until month 0 as the covariate was performed to compare the cumulative total costs after 18 months between both groups. Total costs included outpatient care costs (outpatient costs by homeopathic physicians and outpatient costs by other physicians), medication costs, productivity loss, costs of the integrated care contract, inpatient costs and other costs. Both total costs and single cost types were summarized for the period of 12 months before the study onset (month -12 to month 0) and for the subsequent 18 months, divided into three-month intervals (months 1–3, 4–6, 7–9, 10–12, 13–15, and 16–18) and compared between groups and between diagnoses using ANCOVAs with the respective baseline cost values as a covariate. Cost progression for the groups was compared from both the societal and statutory health insurance perspectives over the observation period. Data on the number of days of sick leave and number of hospital stays were compared between the groups and diagnoses. The test for the primary end point was two-sided with a significance level of 0.05. All other tests were two-sided with a significance level of 0.05, but were exploratory. The number of prescriptions as well as the number of the most common diagnoses (i.e., mental and behavioural, respiratory system, musculoskeletal system and symptoms, signs and abnormal clinical and laboratory findings, not elsewhere classified) were analysed post-hoc and exploratory after discussing the results of the pre-planned analyses.

Matching was performed with SAS software version 9.3 (SAS Institute Inc., Cary, U.S.). We employed a pre-specified statistical analysis plan and used R version 3.1.0 [20] for the data analyses.

## Results

Out of all those insured in this statutory health insurance company, 4,117,726 fulfilled the inclusion criteria. Of those 4,117,726, a sample of ten times more controls than homeopathy patients were drawn at random for each matching process. The matching process was then done in a 1:1 ratio. Data for 44550 patients were included in the analyses, and propensity score matching resulted in 22275 matched pairs. The mean age was 34 years (SD 20.1) and 67.3% of the patients were female (mean age women 35.75, SD 19.00; mean age men 30.51, SD 21.67). The propensity score matching resulted in a comparable sample that showed no relevant differences in baseline characteristics or costs (Table 1).

**Table 1:** Baseline characteristics of all patients. Data include the mean (SD) or the number of persons (%).

	Homeopathy (n = 22275)	Control (n = 22275)
Women (n)	14992 (67.3)	14968 (67.2)
Age (years)	33.85 (20.0)	34.2 (20.1)
Sick leave days previous 12 months	9.58 (35.4)	10.17 (36.4)
Hospital stays previous 12 months	0.23 (0.7)	0.21 (0.6)
Cumulated number of prescriptions previous 12 months (SD)	6.93 (10.5)	7.49 (17.7)
Cumulated costs previous 12 months		
Total	1851 (5365)	1855 (5170)
Medication	304.1 (2884)	318.9 (2453)
Inpatient	556.8 (3057)	554.5 (3142)
Diagnosis (n)		
Other	13479 (60.5)	13479 (60.5)
Mental and behavioural (F)	9131 (41.0)	8652 (38.8)
Depressive disorder (F33)	3072 (13.8)	3072 (13.8)
Migraine (G43)	889 (4.0)	889 (4.0)
Respiratory system (J)	12022 (54.0)	12063 (54.2)
Allergic rhinitis (J30)	1137 (5.1)	1137 (5.1)
Asthma (J45)	1247 (5.6)	1247 (5.6)
Atopic dermatitis (L20)	1488 (6.7)	1488 (6.7)
Musculoskeletal system (M)	10070 (45.2)	10080 (45.3)
Symptoms, signs and abnormal clinical and laboratory findings, not elsewhere classified (R)	10321 (46.3)	10221 (47.4)
Headache (R51)	963 (4.3)	963 (4.3)
State of residence (n)		
Abroad	36 (0.2)	25 (0.1)
Baden-Wuerttemberg	3272 (14.7)	2281 (10.2)
Bavaria	2922 (13.1)	2438 (10.9)
Berlin	2256 (10.1)	1965 (8.8)
Brandenburg	432 (1.9)	546 (2.5)
Bremen	279 (1.3)	172 (0.8)
Hamburg	1274 (5.7)	1042 (4.7)
Hesse	1860 (8.4)	2027 (9.1)
Mecklenburg-Western Pomerania	236 (1.1)	386 (1.7)
Lower Saxony	2115 (9.5)	2326 (10.4)
North-Rhine Westphalia	4162 (18.7)	5812 (26.1)
Rhineland-Palatinate	878 (3.9)	974 (4.4)
Saarland	199 (0.9)	206 (0.9)
Saxony	415 (1.9)	437 (2.0)
Saxony-Anhalt	133 (0.6)	308 (1.4)
Schleswig-Holstein	1512 (6.8)	1046 (4.7)
Thuringia	294 (1.3)	284 (1.3)

The adjusted cumulative total costs 18 months after integrated care contract onset were higher in the homeopathy group than in the control group. The adjusted mean difference of EUR 1350.16 [95% CI 1143.59–1556.73] was statistically significant (homeopathy: adj. mean EUR 7207.72, [7001.14–7414.29]; controls EUR 5857.56 [5650.98–6064.13], p<0.0001). The higher costs in the homeopathy group were primarily driven by productivity loss (homeopathy EUR 3698.00 [3586.48–3809.53]; controls EUR 3092.84 [2981.31–3204.37]; mean difference: 605.16 [584.25–626.07], p<0.0001) and outpatient care costs (homeopathy EUR 1088.25 [1073.90–1102.59]; controls EUR 867.87 [853.52–882.21]; mean difference: 220.38 [212.88–227.88], p<0.0001). Outpatient care costs in the homeopathic group were higher than those in the control group for both components (i.e., both homeopathic treatments and treatments by other physicians) (Table 2). Approximately 50% of the total costs were attributable to productivity loss. However, after productivity loss was subtracted from the total costs, the results indicated a remaining difference of EUR 719.36 [95% CI 562.53–876.19] between the groups. Regarding productivity loss, the cost difference was explained by the difference between the groups in terms of the number of sick leave days after 18 months. Homeopathy users were absent from work an average of 18.77 days over 18 months [95% CI 17.96–19.58], whereas controls had an average of 15.97 sick leave days [15.26–16.67] (p<0.0001) (Table 3). Sick leave days were similar between groups for different diagnoses, except for depression, for which sick leave days were higher in both groups. In patients with depression, the mean number of sick leave days in the homeopathy group was 48.21 [95% CI 44.57–51.84] compared to 41.97 [38.63–45.31] in the control group (p = 0.013). The mean number of hospital stays was 0.48 in the control group [95% CI 0.45–0.52] compared with 0.61 [0.57–0.66] in homeopathy users with depression (p<0.0001).

**Table 2:** Adjusted means for different cost types over 18 months after the start of the integrated care contract for all patients, societal perspective.

	**Homeopathy (n = 22275)**		**Control (n = 22275)**		
Type of cost	N Cost utilization	Adj. mean (EUR) (95% CI)	N Cost utilization	Adj. mean (EUR) (95% CI)	p-value
Integrated care contract	22272	228.00 (226.66–229.35)	–	–	<0.0001
Outpatient	22271	1088.25 (1073.90–1102.59)	22275	867.87 (853.52–882.21)	<0.0001
Homeopathic physician	21658	165.32 (163.45–167.20)	606	25.50 (23.62–27.37)	<0.0001
Other physicians	21802	950.49 (933.17–967.80)	22275	856.37 (839.06–873.69)	<0.0001
Medication	19638	773.27 (656.37–890.17)	20166	579.64 (462.74–696.54)	0.022
Productivity loss	7960	3698.00 (3586.48–3809.53)	7954	3092.84 (2981.31–3204.37)	<0.0001
Inpatient	4702	959.65 (904.08–1015.22)	4334	821.90 (766.33–877.47)	0.001
Other	20604	56.21 (54.54–57.88)	17538	54.64 (52.96–56.31)	0.192
**Total**	**22275**	**7207.72 (7001.14–7414.29)**	**22275**	**5857.56 (5650.98–6064.13)**	**<0.0001**

**Table 3:** Mean cumulative sick leave days, number of hospital stays, diagnoses and prescriptions over 18 months after the start of the integrated care contract for different diagnoses.

	Homeopathy		Control		
Variable	N utilization	Mean (95% CI)	N utilization	Mean (95% CI)	p-value
Sick leave days	7960	18.77 (17.96–19.58)	7954	15.97 (15.26–16.67)	<0.0001
Number of hospital stays	4709	0.36 (0.34–0.37)	4344	0.31 (0.30–0.32)	<0.0001
Cumulative number of diagnoses					
Mental and behavioural (F)	13450	0.60 (0.60–0.61)	9774	0.44 (0.43–0.45)	0.03
Respiratory system (J)	15385	0.69 (0.68–0.70)	14096	0.63 (0.63–0.64)	<0.0001
Musculoskeletal system (M)	13175	0.59 (0.59–0.60)	11956	0.54 (0.53–0.54)	0.033
Symptoms, signs and abnormal clinical and laboratory findings, not elsewhere classified (R)	14978	0.67 (0.67–0.68)	12254	0.55 (0.54–0.56)	<0.0001
Number of prescriptions	19851	10.40 (10.2–10.6)	20233	10.69 (10.5–10.9)	0.034

Regarding disease-specific costs, patients with depression accounted for most of these costs. Among the group suffering from depression, the adjusted cumulative total costs were higher in the homeopathy group than in the control group (homeopathy adj. mean: EUR 15084.49, [95% CI 14460.27–15708.71]; controls EUR 12797.66 [12173.44–13421.88], p<0.0001) (Table 4). For the breakdown of total costs in specific diagnosis subgroups, see S1 Table.

**Table 4:** Adjusted means for total costs for different diagnoses over 18 months after the start of the integrated care contract for all patients, societal perspective.

	Homeopathy		Control		
Diagnosis	N Cost utilization	Adj. mean (EUR) (95% CI)	N Cost utilization	Adj. mean (EUR) (95% CI)	p-value
All	22275	7207.72 (7001.14–7414.29)	22275	5857.56 (5650.98–6064.13)	<0.0001
Depression	3072	15084.49 (14460.27–15708.71)	3072	12797.66 (12173.44–13421.88)	<0.0001
Migraine	889	8512.25 (7644.90–9379.59)	889	7115.62 (6248.27–7982.97)	0.026
Allergic rhinitis	1137	5763.05 (5252.59–6273.51)	1137	4642.93 (4132.47–5153.39)	0.002
Asthma	1247	6937.04 (6351.49–7522.60)	1247	5541.33 (4955.77–6126.88)	0.001
Atopic dermatitis	1488	4256.71 (3922.12–4591.31)	1488	3426.10 (3091.50–3760.70)	0.001
Headache	963	7597.46 (6783.55–8411.37)	963	6279.82 (5465.90–7093.73)	0.025

**Fig 1:**
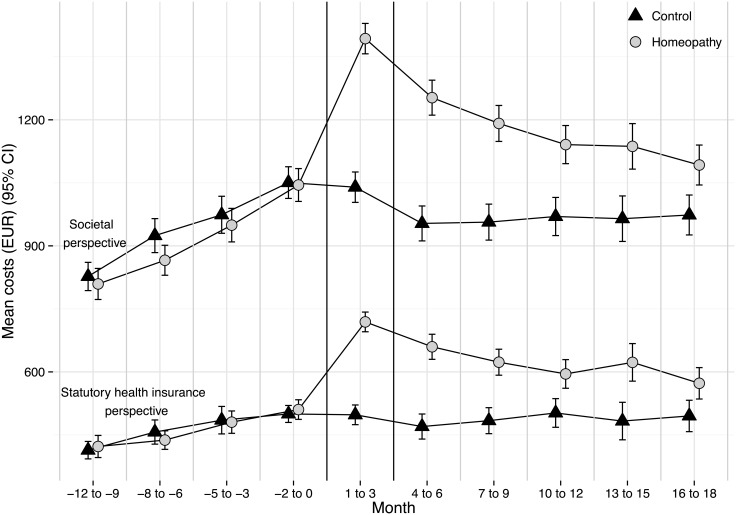
Mean overall cost (EUR) progression by group from the societal and statutory health insurance perspectives from month -12 until month 18. Error bars denote 95% CIs. Months 1 to 3 indicate the start of the integrated care model. Costs from month 1 on adjusted to baseline costs (month -12 to month 0)

From both the societal and statutory health insurance perspectives, the cost progression was similar in both groups from month -12 to month 0 (cumulative costs from month -12 to month 0 from the societal perspective: homeopathy mean EUR 3669 [95% CI 3553–3785]; controls mean EUR 3776 [3662–3890]; statutory health insurance: homeopathy mean EUR 1851 [1780–1921]; controls mean EUR 1855 [1788–1923]). The greatest cost difference could be observed in months 1–3 (societal perspective: homeopathy mean EUR 1382 [1336–1429]; controls mean EUR 1051 [1011–1090]; statutory health insurance perspective: homeopathy mean EUR 718.5 [687.6–749.3]; controls mean EUR 498.3 [474.3–522.4]). This substantial difference largely resulted from the costs for initial homeopathic consultations. Costs progressed similarly from both the societal and statutory health insurance perspectives. From both perspectives, the costs for the homeopathy group were higher than those for the control group for the entire observation period. However, by the end of the observation period (month 18), the gap between the two groups had decreased (Fig 1). The number of prescriptions for homeopathic patients was lower than that for control patients after 18 months (homeopathy mean 10.4 [95% CI 10.2–10.6]; controls mean 10.7 [10.5–10.9], p = 0.038). The costs for medication after 18 months differed between the groups (homeopathy adj. mean EUR 773.27 [95% CI 656.37–890.17]; controls EUR 579.64 [462.74–696.54], p = 0.022). After 18 months, the mean number of diagnoses was higher in the homeopathic group than in the control group for all observed diagnoses (Table 3).

## Discussion

### Key results

Our analysis showed that adjusted total health care costs after 18 months across diagnoses were higher in the homeopathy group compared to the control group. The cost difference was primarily attributable to productivity loss and outpatient care costs. Group differences decreased over time.

### Strengths and limitations

This study used claims data from a large statutory health insurance company for secondary data analyses, resulting in a sample size of 44550 patients, including 22275 subscribers to the integrated care contract homeopathy. We used cumulative different unit costs for 12 months prior to the integrated care contract as a covariate for the propensity score. The nature of secondary data used in our study does not allow to draw conclusions about the severity of the patients’ diseases. However, health care costs can serve as a proxy for a medical condition or for the severity of a disease [21]. Therefore we reduced the variances of unknown variables responsible for the cost differences between the two groups. The matching resulted in comparable groups with respect to the considered covariates, confirmed by both the analysis at baseline and the pre-baseline cost progression analyses. We analysed a number of types of costs, including productivity loss, and were therefore able to consider costs from both the payer and societal perspectives.

A limitation of our study is that it was restricted to costs; therefore, we were unable to evaluate the effectiveness of the integrated care contract. Approximately 40% of the patients were matched according to a confirmed diagnosis. However, the patients’ reasons, primary or otherwise, for visiting a physician during the first three months of the observation period were unknown. For example, two patients could have been matched for the diagnosis of asthma although one of the matches might have also experienced and sought treatment for a costly car accident. However, such a situation could have occurred in both groups; because the matching also involved costs, we assume that we balanced the burden of disease in both groups. Another limitation of our study is the relatively short observation period of 18 months. Given that the costs for both groups appeared to converge at the end of the observation period, a longer observation period would have been desirable. Homeopathic treatment could be associated with initial higher investments that decline over longer courses of treatment. However, the data available for our study did not allow for testing this assumption. Regarding the generalizability of our results, approximately two-thirds of our sample comprised female participants, reflecting the current literature on predictions for complementary medicine usage [22]. Furthermore, the generalizability of our results is limited because we used data from only one statutory health insurance company. Historically, the clients of this health insurance company are younger and better educated than clients from other health insurance companies. Moreover, patients who subscribe to the integrated care contract may differ from patients who do not subscribe in terms of unobservable factors, such as lifestyle factors or health consciousness. Unfortunately, these factors were unknown to us, and we could not adjust for them. Another limitation is that neither homeopathic remedies nor over-the-counter drugs paid for out of pocket could be considered in the analyses from the societal perspective. This limitation might have resulted in an underestimation of costs for both groups.

### Interpretation

A recent systematic review by Viksveen on the cost-effectiveness of homeopathy showed that in eight out of fourteen studies, the homeopathic treatment was less cost-intensive than the conventional treatment; in four studies, the treatment costs were similar; and in two studies, the homeopathic treatment was more costly than conventional treatment [7]. However, the review also indicated that the included economic evaluations of homeopathy were heterogeneous and lacked methodological quality. A recent Dutch secondary data analysis compared health care costs for patients treated by a conventional GP compared with patients treated by a GP trained in complementary and alternative medicine (CAM) for six years. The researchers reported that the annual health care costs for patients treated by a CAM-GP were nearly EUR 200 lower than the costs for patients treated by a conventional GP [14]. Unfortunately, the authors did not present subgroup analyses for different types of CAM. In their analyses, the researchers did not adjust for baseline costs and did not explore comorbidities or longitudinal cost progressions. Overall, the literature on the costs of homeopathic treatment has not reached a clear conclusion. Furthermore, because of differences in health care systems, the transferability of health economics results from one country to another is limited [23]. Therefore, our study adds important knowledge to this body of literature.

The Viksveen review reported that the most relevant cost driver in homeopathic treatment are consultation costs and that overall medication costs are often reduced for homeopathic treatment compared with conventional practices [7]. By contrast, our results showed that a cost increase for homeopathic patients could be observed for many types of costs, including costs for medications, outpatient care, and productivity loss. For the increase in conventional medication costs that we observed, two explanations might be applicable: either homeopathic physicians who were also conventionally trained prescribed them, or patients sought more conventional care from other physicians. Detailed post hoc analyses of prescriptions showed that although homeopathic patients had higher medication costs, the number of prescriptions was similar in both groups. Homeopathic patients might therefore have been prescribed higher-priced medications. Interestingly, homeopathic patients received three-quarters of their conventional drug prescriptions from other physicians. Many patients seeking treatment from homeopaths are chronically ill [24]. Patients under integrated care contracts might be better integrated into the health system than other chronically ill patients. In general, homeopathic treatment follows a more holistic approach that considers the whole person and his or her resources. This approach could result in diagnosing and treating other somatic or mental disorders as well and in more frequently providing patients with sick leave notes allowing them to rest. This more holistic approach could lead to the initiation of further conventional care, which is associated with additional costs. This possible situation might explain why the homeopathic group had more diagnoses after 18 months, with a peak at the beginning of the integrated care contract, although the numbers of diagnoses were comparable between the groups at baseline. In months 1–3, considering all diagnosis groups, homeopathic patients had 126.2% more diagnoses than the controls did. The greatest difference between the groups was observed for mental health diagnoses (38.9%). The difference in the observed number of diagnoses after the beginning of the integrated care contract might be explained by some patients who visited the homeopathic physician for the first time and the associated extensive initial consultation including broader diagnostics. This could have been a reason for higher cost. However, we do not have any data available to support that claim. Another explanation for the difference in the number of diagnoses might be that homeopathic physicians who participated in the integrated care contract might have provided more thorough documentation even for their own patients because of the new contact format, thus resulting in more diagnoses.

### Conclusion

The analyses of our present study did not confirm the previously observed cost savings resulting from the use of homeopathy in the health care system. In an 18-month integrated care program that offered and reimbursed homeopathy in addition to usual care, patients who used additional homeopathic treatment had significantly higher costs compared with patients who received only usual care.

## Supporting Information

S1 Table Adjusted means for different cost types and diagnoses over 18 months after the beginning of the integrated care contract, societal perspective. (PDF)
