# Supplementary material for: Can Additional Homeopathic Treatment Save Costs? A Retrospective Cost-Analysis Based on 44500 Insured Persons
Source: PLoS One. 2015 Jul 31;10(7):e0134657. doi: 10.1371/journal.pone.0134657 (PMC4521756; doi:10.1371/journal.pone.0134657)
Supplement: S1 Table — (PDF) [file pone.0134657.s001.pdf]

S1 Table. Adjusted means for different cost types and diagnoses over 18 months after the beginning of the integrated care contract, societal perspective

		Homeopathy		Control		
	Type of cost	N Cost utilization	Adj. mean (EUR) (95% CI)	N Cost utilization	Adj. mean (EUR) (95% CI)	p-value
**All n=44550**	Integrated care contract	22272	228.00 (226.66–229.35)	–	–	<0.0001
	Outpatient	22271	1088.25 (1073.90–1102.59)	22275	867.87 (853.52–882.21)	<0.0001
	Homeopathic physician	21658	165.32 (163.45–167.20)	606	25.50 (23.62–27.37)	<0.0001
	Other physicians	21802	950.49 (933.17–967.80)	22275	856.37 (839.06–873.69)	<0.0001
	Medication	19638	773.27 (656.37–890.17)	20166	579.64 (462.74–696.54)	0.022
	Productivity loss	7960	3698.00 (3586.48–3809.53)	7954	3092.84 (2981.31–3204.37)	<0.0001
	Inpatient	4702	959.65 (904.08–1015.22)	4334	821.90 (766.33–877.47)	0.001
	Other	20604	56.21 (54.54–57.88)	17538	54.64 (52.96–56.31)	0.192
	**Total**	**22275**	**7207.72 (7001.14–7414.29)**	**22275**	**5857.56 (5650.98–6064.13)**	**<0.0001**
**Depression n=6144**	Integrated care contract	3072	272.67 (268.52–276.81)	–	–	<0.0001
	Outpatient	3072	2023.03 (1973.21–2072.86)	3072	1624.87 (1575.05–1674.70)	<0.0001
	Homeopathic physician	2977	212.33 (204.31–220.34)	111	43.45 (35.43–51.46)	<0.0001
	Other physicians	3061	1808.88 (1759.62–1858.14)	3072	1601.61 (1552.35–1650.86)	<0.0001
	Medication	2869	1146.34 (1014.67–1278.02)	2952	1084.14 (952.46–1215.81)	0.513
	Productivity loss	1696	9039.87 (8548.30–9531.45)	1593	7616.13 (7124.56–8107.71)	0.0001
	Inpatient	997	1787.79 (1619.45–1956.13)	888	1561.24 (1392.90–1729.58)	0.062
	Other	4083	103.26 (96.90–109.62)	3501	98.73 (92.37–105.09)	0.324
	**Total**	**3072**	**15084.49 (14460.27–15708.71)**	**3072**	**12797.66 (12173.44–13421.88)**	**<0.0001**
**Migraine n=1778**	Integrated care contract	889	248.65 (242.01–255.30)	–	–	<0.0001
	Outpatient	888	1191.03 (1123.33–1258.73)	889	994.28 (926.59–1061.98)	0.0001
	Homeopathic physician	858	171.98 (163.92–180.04)	23	24.21 (16.15–32.27)	<0.0001
	Other physicians	882	1025.20 (958.10–1092.30)	889	978.68 (911.58–1045.78)	0.337
	Medication	803	529.76 (420.44–639.08)	838	554.77 (445.45–664.10)	0.751
	Productivity loss	467	4945.54 (4324.02–5567.07)	458	4062.14 (3440.61–4683.66)	0.049
	Inpatient	213	1173.94 (816.94–1530.93)	191	1081.87 (724.88–1438.87)	0.721
	Other	963	50.43 (44.11–56.76)	833	46.57 (40.24–52.89)	0.397
	**Total**	**889**	**8512.25 (7644.90–9379.59)**	**889**	**7115.62 (6248.27–7982.97)**	**0.026**
**All. Rhinitis n=2274**	Integrated care contract	1137	243.76 (237.87–249.64)	–	–	<0.0001
	Outpatient	1137	979.76 (933.65–1025.86)	1137	783.78 (737.67–829.88)	<0.0001
	Homeopathic physician	1102	163.95 (156.90–171.00)	36	20.58 (13.53–27.63)	<0.0001
	Other physicians	1115	820.34 (774.81–865.86)	1137	766.60 (721.08–812.13)	0.102
	Medication	1005	574.59 (437.06–712.13)	1054	449.92 (312.39–587.46)	0.209
	Productivity loss	448	3025.98 (2634.78–3417.17)	447	2528.19 (2137.00–2919.38)	0.078
	Inpatient	193	586.16 (472.07–700.25)	212	501.47 (387.38–615.57)	0.304
	Other	1048	42.99 (35.79–50.20)	880	40.31 (33.10–47.51)	0.605
	**Total**	**1137**	**5763.05 (5252.59–6273.51)**	**1137**	**4642.93 (4132.47–5153.39)**	**0.002**
**Asthma n=2494**	Integrated care contract	1247	239.19 (233.20–245.18)	–	–	<0.0001
	Outpatient	1247	1114.48 (1043.70–1185.25)	1247	940.38 (869.60–1011.15)	0.001
	Homeopathic physician	1210	166.79 (158.42–175.16)	27	36.63 (28.26–45.00)	<0.0001
	Other physicians	1236	951.34 (844.67–1058.02)	1247	948.83 (842.16–1055.50)	0.974
	Medication	1191	866.57 (742.54–990.60)	1208	656.18 (532.15–780.21)	0.019
	Productivity loss	430	3241.45 (2845.82–3637.08)	427	2550.37 (2154.75–2946.00)	0.016
	Inpatient	283	1026.66 (819.48–1233.84)	267	896.91 (689.73–1104.09)	0.385
	Other	1201	52.87 (45.91–59.82)	1051	56.71 (49.76–63.67)	0.443
	**Total**	**1247**	**6937.04 (6351.49–7522.60)**	**1247**	**5541.33 (4955.77–6126.88)**	**0.001**
**Atopic dermatitis n=976**	Integrated care contract	1488	218.26 (213.34–223.18)	–	–	<0.0001
	Outpatient	1488	917.18 (863.78–970.58)	1488	702.63 (649.23–756.03)	<0.0001
	Homeopathic physician	1456	153.47 (148.75–158.19)	41	22.86 (18.14–27.58)	<0.0001
	Other physicians	1471	797.39 (715.28–879.50)	1488	678.52 (596.41–760.63)	0.045
	Medication	1393	439.42 (372.41–506.43)	1417	405.63 (338.62–472.64)	0.485
	Productivity loss	379	1763.88 (1533.07–1994.69)	339	1412.06 (1181.25–1642.87)	0.035
	Inpatient	279	570.51 (461.08–679.93)	242	502.47 (393.04–611.89)	0.389
	Other	1202	43.52 (39.22–47.83)	1037	45.39 (41.09–49.70)	0.547
	**Total**	**1488**	**4256.71 (3922.12–4591.31)**	**1488**	**3426.10 (3091.50–3760.70)**	**0.001**
**Headache n=1926**	Integrated care contract	963	224.18 (217.91–230.46)	–	–	<0.0001
	Outpatient	963	1177.79 (1113.59–1241.99)	963	879.65 (815.45–943.85)	<0.0001
	Homeopathic physician	933	151.41 (144.29–158.53)	20	24.98 (17.86–32.10)	<0.0001
	Other physicians	955	1032.15 (968.29–1096.01)	963	857.33 (793.47–921.20)	0.0002
	Medication	870	570.22 (434.88–705.56)	897	486.45 (351.11–621.80)	0.391
	Productivity loss	366	4459.68 (3856.08–5063.27)	360	3471.35 (2867.75–4074.94)	0.023
	Inpatient	224	826.94 (561.27–1092.62)	213	936.77 (671.10–1202.45)	0.567
	Other	1016	50.27 (44.98–55.55)	852	46.31 (41.03–51.60)	0.299
	**Total**	**963**	**7597.46 (6783.55–8411.37)**	**963**	**6279.82 (5465.90–7093.73)**	**0.025**
